# Hypereosinophilic syndrome with cardiac and cutaneous involvement: Missed treatment opportunity of a case from Ethiopia

**DOI:** 10.1002/ccr3.8844

**Published:** 2024-05-21

**Authors:** Kedir Negesso Tukeni, Tadesse Dukessa Gemechu, Mohammed Abafogi, Amare Ashine, Tamirat Godebo Woyimo, Abdo Abafogi, Elsah Tegene Asefa

**Affiliations:** ^1^ Jimma University Jimma Ethiopia

**Keywords:** biventricular thrombus, Ethiopia, hypereosinophilia, Jimma Medical Center, myocarditis

## Abstract

Understand the importance of considering alternative diagnosis in patients presenting with atypical features. Understand the importance of considering common presentations of rare cases. Underscoring the critical importance of timely recognition and appropriate management of potentially life‐threatening condition.

## 1 INTRODUCTION

Hypereosinophilic syndrome is a rare disorder characterized by sustained eosinophilia for at least six consecutive months, associated with organ damage attributable to tissue hypereosinophilia. The clinical presentation varies depending on the organs affected but commonly involves skin, heart, lungs, gastrointestinal tract, and central nervous system. Here we report the case of 52‐year‐old patient who developed idiopathic hypereosinophilic syndrome with cardiac and cutaneous involvement, biventricular thrombus, and left leg gangrene. Due to the disease's rarity and the length of time it took for investigations to be completed, the case was lost as the heart failure and infection therapy were primarily advised.

## 2 CASE PRESENTATION

A 52‐year‐old Black man who did not smoke was presented for evaluation of his worsening cough, dyspnea, orthopnea, paroxysmal nocturnal dyspnea, body swelling that began in his legs, and the formation of skin lesion on his left leg that subsequently ulcerated. He has had intermittent dry cough for a year; it is worst at night and gets worse when he walks long distances. He did not seek medical assistance as he thought getting some rest would help. This could also be due to insufficient health care facilities, financial restraints, or cultural beliefs that things would better after a particular amount of time. As a result, he did not have a clear diagnosis and hence did not receive effective management previously.

Otherwise, he had no history of psychiatric issues, drug allergies, chicken pox, mumps, smallpox, or any other childhood illnesses. He was raised in Ethiopia's Oromia region, where he was born. Though he never attended school, he is able to read and write numbers. He is a father of nine children.

**FIGURE 1:**
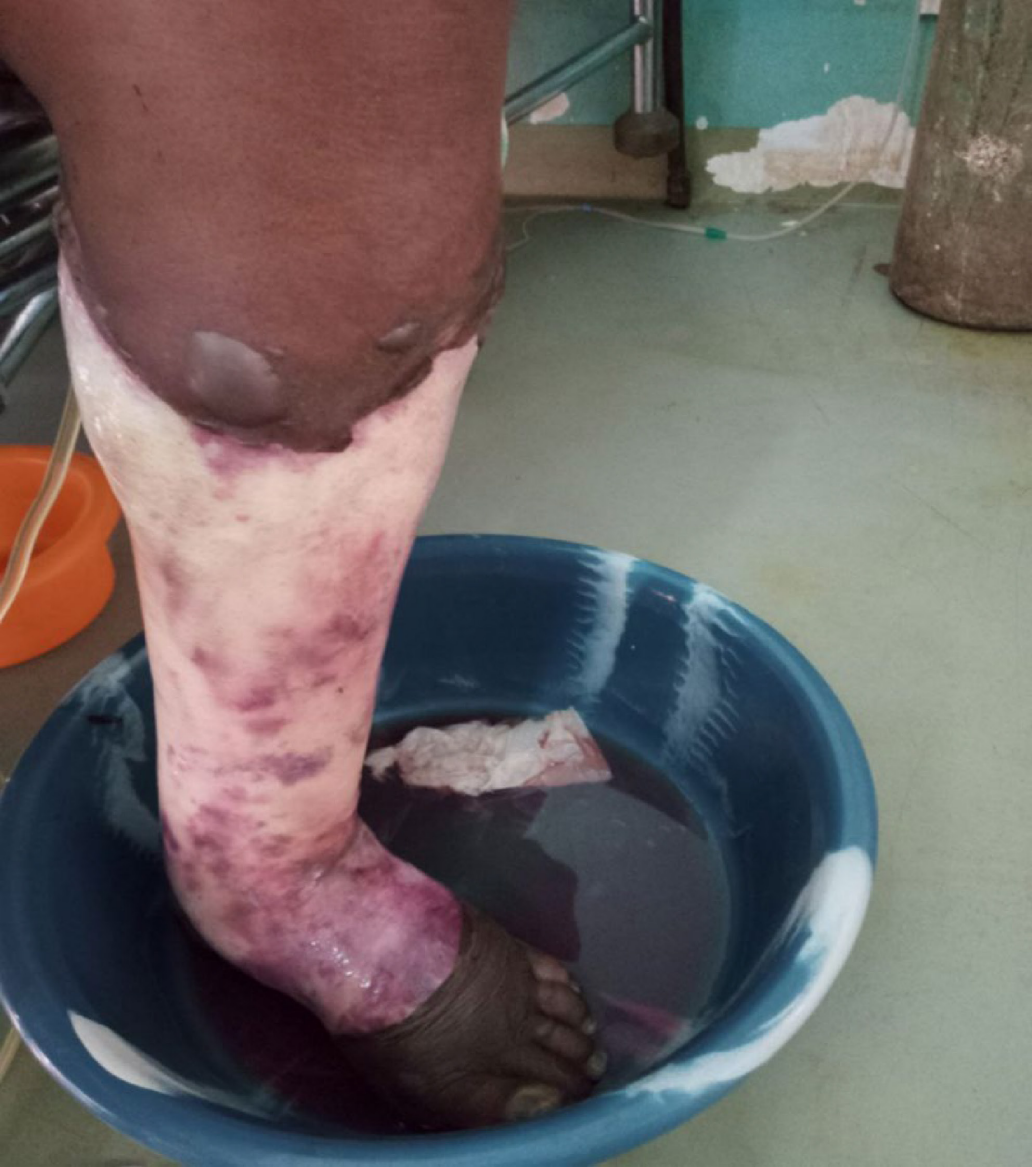
Erythematous leg with blisters with extensive skin excoriation, adjacent skin showing dark discoloration and watery discharge suggestive of Stage 4 necrotizing fasciitis.

Upon evaluation, the patient was in cardiorespiratory distress with severe pain on the left leg. His blood pressure was 95/60 mmHg; pulse rate of 112 beats per minute, feeble; breathing rate of 30 breaths per minute; temperature was 36.7°C and saturation of 89% with room air. Breath sound was less audible over the left infrascapular region. The jugular venus pulse was elevated 5 cm above the sternal angle, and there was Grade III holosystolic murmur best heard at apex. There was evidence of fluid collection in abdomen. The left leg was hot, reddish, and swollen, with surrounding areas of dark discoloration (Figure 1).

### 2.1 Differential diagnosis

Infections, parasitosis, and malignant illnesses are among the differential diagnoses for hypereosinophilia. In as many as 13% of individuals, eosinophilic bronchitis is the common cause of chronic cough. However, because it is not linked to peripheral blood eosinophilia, eosinophilic bronchitis differs significantly from HES.^1^, ^2^ While eosinophilic leukemia is included in the differential diagnosis of HES, we excluded it in our case due to the patient's long surviving period—8 years!

### 2.2 Investigation and management

**FIGURE 2:**
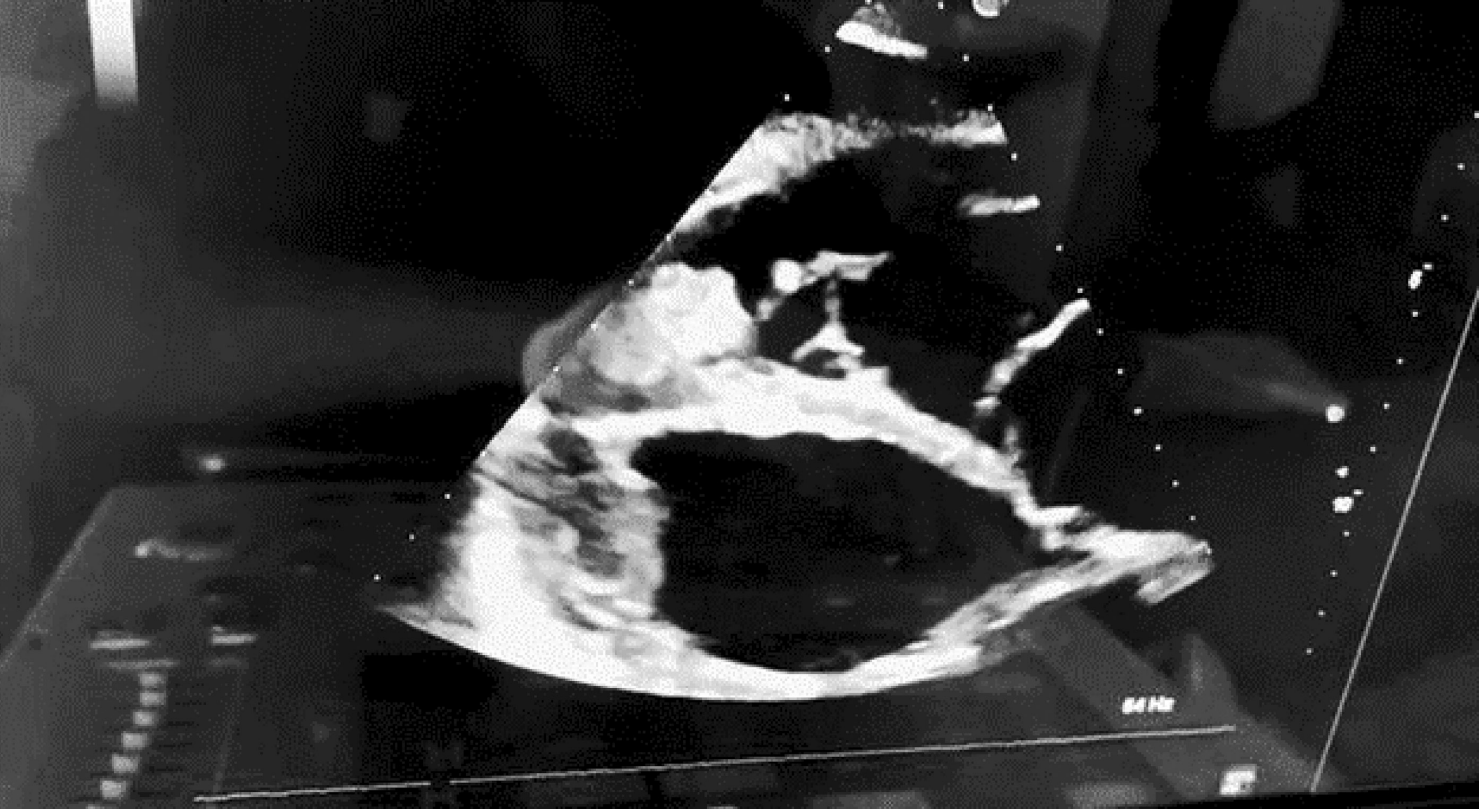
Echocardiogram of the patient showing bilateral ventricular thrombus.

**FIGURE 3:**
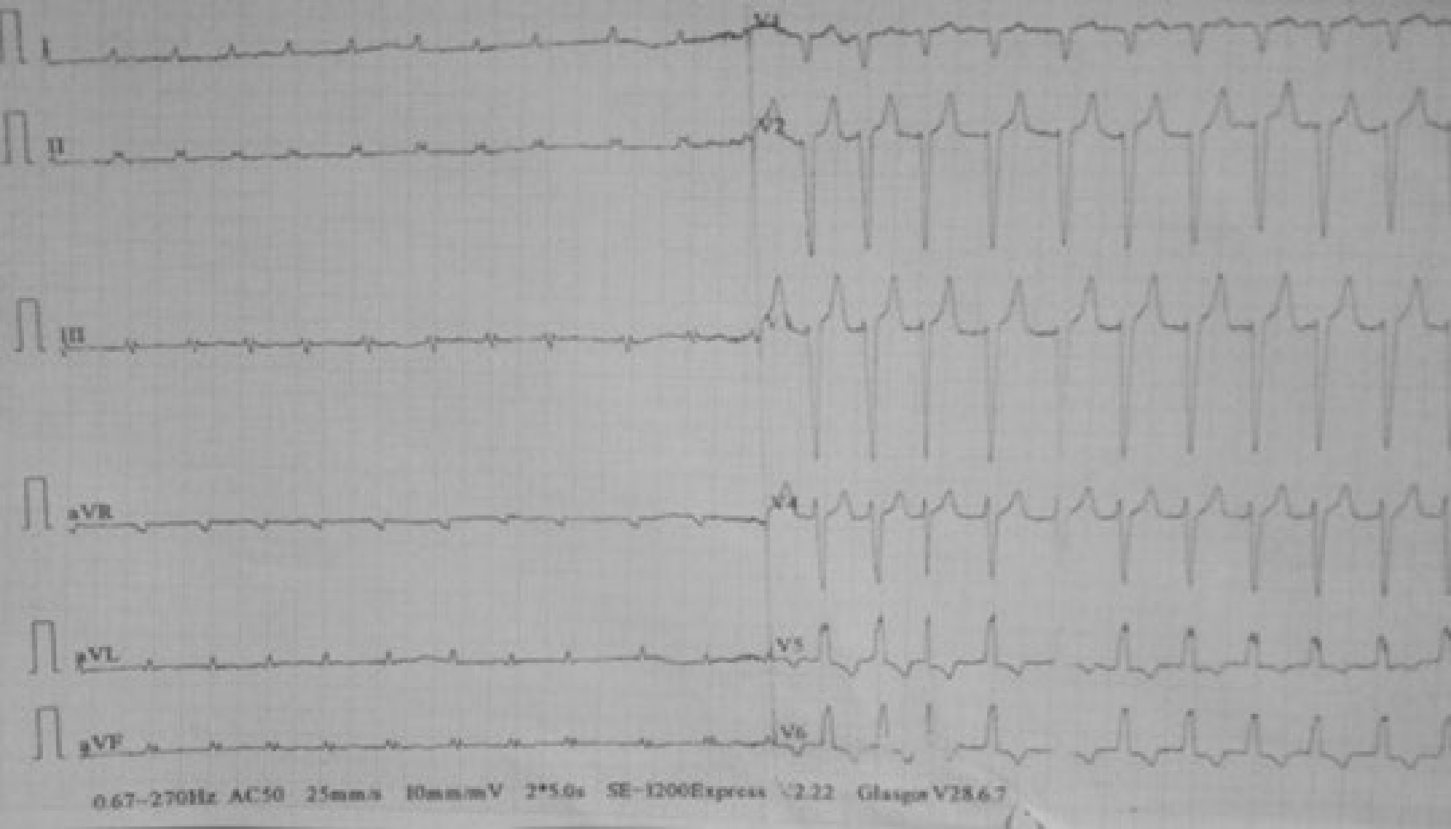
Electrocardiogram of the patient showing atrial fibrillation with heart rate of 142–150 beat per minute and hyperacute T waves.

A complete blood count showed 18,000 WBC/mm^3^, eosinophilia of 13% with absolute eosinophilic count of 2340 cells/mm3, hemoglobin of 18.7 g/dL, and platelet of 39,000/mm^3^. Cardiac troponin increased six times from the baseline and CRP was increased twenty times. The serum electrolytes showed potassium of 6.9 mmol/L, sodium 116 mmol/L, while calcium levels were normal. Coagulation profile, renal and liver function tests, stool exam, and urinalysis were normal. Abdominal ultrasound revealed fluid collection. PA chest x ray revealed Grade I pulmonary edema, Transthoracic echocardiography showed hypokinetic dilated ventricles with impaired systolic function, thrombi in both ventricular apexes (Figure 2 and Video S1), while electrocardiogram showed low voltages, left bundle branch block, and atrial fibrillation with a fast ventricular response (Figure 3).

**FIGURE 4:**
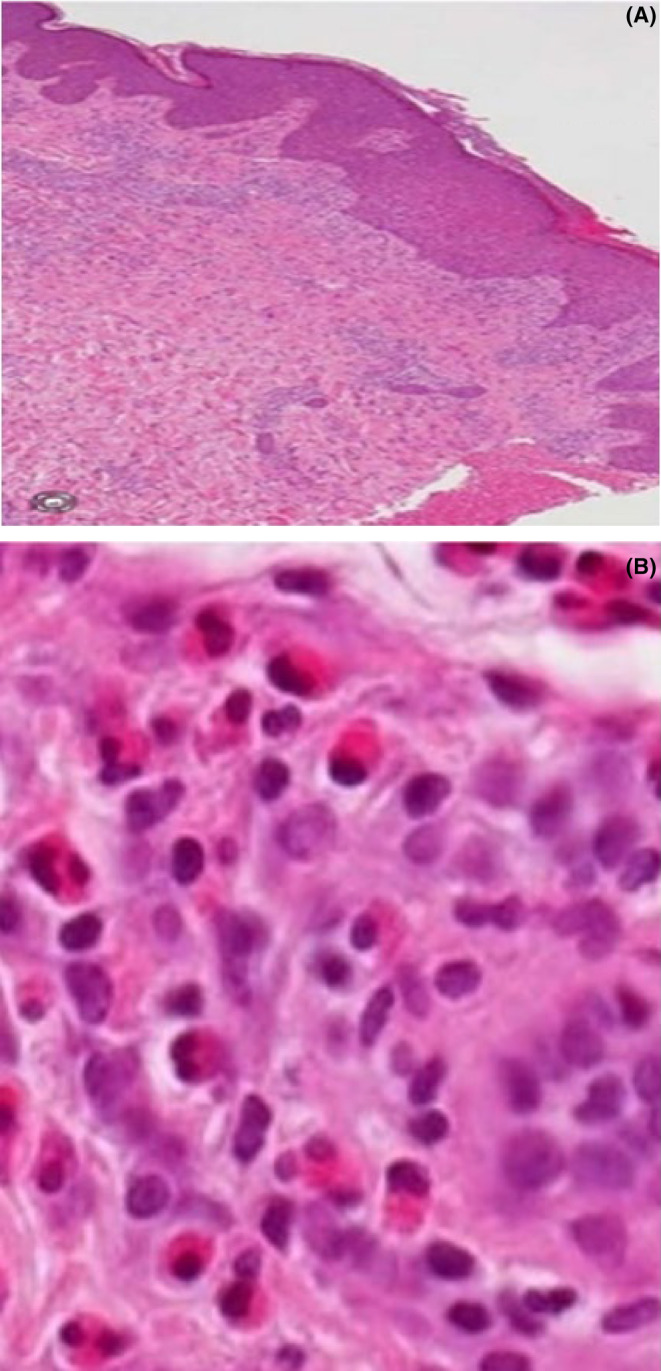
(A, B) Within the dermis, there is a moderate perivascular mixed inflammatory infiltrate with sheets of eosinophils, the overlying epidermis shows acanthosis and hyperkeratosis.

The skin biopsy of the patient showing dermal moderate perivascular mixed inflammatory infiltrate with sheets of eosinophils, the overlying epidermis shows acanthosis and hyperkeratosis (Figure 4). (Note that the result was collected after the patient passed away).

In light of the aforementioned data, the possibility of hypereosinophilic syndrome affecting the skin and heart (myocarditis) was explored. He was started on anticoagulation, ant‐ischemic, antibiotics, and supportive care despite a delay for initiation of steroid while waiting for biopsy results. Even though the patient was not suitable for surgery, the surgical team was approached for necrotizing fasciitis.

## 3 RESULTS AND FOLLOW UP

The patient gradually developed mixed septic shock, and cardiogenic shock. Sadly, he passed away 2 days after being admitted to the hospital, with the possible causes of death being multiorgan failure due to mixed septic and cardiogenic shock.

## 4 DISCUSSION

HES is a rare disorder characterized by sustained eosinophilia (>1.5 × 10^9/L^) for at least six consecutive months, associated with organ damage attributable to tissue hypereosinophilia.^3^, ^4^ The etiology of HES remains unknown, but believed to involve complex interactions between genetic predisposition, dysregulated immune responses, and environmental triggers.^3^, ^5^

The clinical presentation varies depending on the organs affected by eosinophilic infiltration. Commonly involved organs include skin, heart, lungs, gastrointestinal tract, and central nervous system.^3^ Cardiovascular complications account for significant HES‐related morbidity and mortality.^6^, ^7^, ^8^, ^9^ In addition to direct eosinophil‐mediated damage to the heart, coronary vasospasm, arterial and/or venous thrombi as well as microvascular damage may occur.^8^, ^10^

Diagnosis of HES requires a high index of suspicion and careful evaluation to exclude secondary causes of hypereosinophilia. Laboratory investigations reveal elevated eosinophils in peripheral blood, with evidence of end‐organ damage on imaging studies or tissue biopsy. Histopathological examination of affected tissues may demonstrate eosinophilic infiltration, confirming the diagnosis.^11^ Treatment options include corticosteroids, cytotoxic agents, targeted biologic therapies, and stem cell transplantation in selected cases. Regular monitoring of eosinophil counts and organ function is essential to assess treatment response.

This case is noteworthy as it satisfies the majority of primary HES criteria; as it involves a patient's cutaneous skin lesion, myocarditis, and biventricular thrombus. The left leg involvement, which mimics peripheral vascular disease with significant limb ischemia, makes this case exceptional as well. Hardy and Anderson originally described HES in 1968.^12^, ^13^ White men are affected more frequently than women in documented instances; however, the severity of the disease is the same in both sexes.^14^ While any organ system may be affected, the most common causes of disease and mortality are neurologic and cardiopulmonary failure. Myocardial infarction, restrictive cardiomyopathy, left or right ventricular insufficiency, and endomyocardial fibrosis are the most severe involvements. Endomyocardial biopsy is the gold standard for the diagnosis but associated with a high morbidity and mortality.^10^, ^13^, ^14^ Instead, echocardiography was used for the evaluation of our patient.

Eosinophil sequestration in organ tissues is the fundamental pathophysiology of HES. The endothelium is damaged by eosinophil‐derived neurotoxic, eosinophil cationic protein, and major basic protein, which are secreted by eosinophils and lead to fibrosis, thrombosis, and infarction.^13^, ^14^ Up to 60% of cases have pulmonary involvement; often have persistent, chronic cough that may mimic asthma. However, the lung function tests usually show no restriction in airflow. An intrinsic eosinophilic infiltration of the lung or emboli originating from right ventricular thrombi may also be the cause of pulmonary involvement.^1^, ^2^, ^13^, ^14^ As there was no sign of pulmonary embolism, heart failure was identified as the origin of the patient's pulmonary symptoms in our case.

HES frequently manifests neuropsychiatric symptoms like cognitive decline, depression, and impaired motor skills as the central nervous system is involved. However, our patient did not have obvious neuropsychiatric abnormalities. Scleritis, keratoconjunctivitis sicca, and Adie's syndrome (pupillotonia) are the primary ocular involvement conditions.^13^ Hyperemic conjunctivas and scleritis did not affect our patient.

Regarding treatment of these patients, some HES patients with pulmonary embolism or other thrombotic processes benefit from anticoagulant therapy, but others advise against it because it does little to stop more thrombosis.^13^, ^14^ As the patient in our case exhibited biventricular thrombi with potentially distant showering of the thrombus endangering the limbs, we suggested anticoagulation along with other heart failure treatments. The patient's condition rapidly deteriorated due to a delay in considering the diagnosis, which left little time for appropriate management.

### 4.1 Limitations

While the management team awaited the biopsy results, the start of steroids was postponed. Furthermore, the patient's quick decline made it difficult for the team to use the entire setup and all of its resources to manage the patient to the optimum.

## 5 CONCLUSION

Hypereosinophilic syndrome is a rare disorder characterized by persistent eosinophilia and multiorgan involvement. This case highlights the complexities involved in diagnosing and treating HES, particularly in resource‐limited settings like Ethiopia. The patient's condition was initially misdiagnosed, underscoring the critical importance of timely recognition and appropriate management of this potentially life‐threatening condition.

## AUTHOR CONTRIBUTIONS

**Kedir Negesso Tukeni:** Conceptualization; data curation; formal analysis; investigation; supervision; validation; writing – original draft. **Tadesse Dukessa Gemechu:** Conceptualization; data curation; investigation; supervision. **Mohammed Abafogi:** Conceptualization; investigation; methodology; visualization. **Amare Ashine:** Investigation; writing – original draft. **Tamirat Godebo Woyimo:** Investigation; software; writing – original draft. **Abdo Abafogi:** Conceptualization; formal analysis; investigation; resources. **Elsah Tegene Asefa:** Conceptualization; data curation; formal analysis; supervision; writing – original draft.

## FUNDING INFORMATION

The authors declare that they are not associated with any organizations, nor do they receive funding from them that could be pertinent to the data in this manuscript preparation.

## ETHICAL STATEMENT

Ethical clearance was not needed in writing of this case summary, however, information gathered from the record and investigation was kept confidential by not writing patient's name and identification of the patient in any way.

## CONSENT

Written informed consent was obtained from the patient's family to publish this report in accordance with the journal's patient consent policy.

## Supporting information


Video S1.

## Data Availability

The data that supports the findings of this study are available in the main manuscript of this article.
